# Rilpivirine use in the Swiss HIV cohort study: a prospective cohort study

**DOI:** 10.1186/s12879-017-2579-2

**Published:** 2017-07-06

**Authors:** Delphine Sculier, Angèle Gayet-Ageron, Manuel Battegay, Matthias Cavassini, Jan Fehr, Cedric Hirzel, Patrick Schmid, Enos Bernasconi, Alexandra Calmy, for the Swiss HIV Cohort Study

**Affiliations:** 1 Division of Infectious Diseases, University Hospital Geneva, Geneva, Switzerland; 2 Clinical Research Center and Division of Clinical Epidemiology, Department of Health and Community Medicine, University Hospital Geneva, Geneva, Switzerland; 3 Division of Infectious Diseases and Hospital Epidemiology, University Hospital Basel, Basel, Switzerland; 4 Division of Infectious Diseases, University Hospital of Lausanne, Lausanne, Switzerland; 5 Division of Infectious Diseases and Hospital Epidemiology, University Hospital Zurich, University of Zurich, Zurich, Switzerland; 6 University Clinic of Infectious Diseases, University Hospital Bern, Bern, Switzerland; 7 Division of Infectious Diseases and Hospital Epidemiology, St. Gallen Cantonal Hospital, St. Gallen, Switzerland; 8 Division of Infectious Diseases, Ospedale Regionale, Lugano, Switzerland

**Keywords:** HIV-1, Rilpivirine, First-line regimen, Treatment simplification, Virological response, Safety

## Abstract

**Background:**

Rilpivirine is safe and effective in HIV-naïve patients with low baseline HIV-RNA or in switch strategy. It offers the advantages of few drug-drug interactions and a favourable toxicity profile. We aimed to determine the reasons for prescribing the rilpivirine (RPV)/tenofovir disoproxil (TDF)/emtricitabine (FTC) co-formulation within the Swiss HIV Cohort Study and to assess its effectiveness and safety over a 24 months period.

**Methods:**

All individuals enrolled in the Swiss HIV Cohort Study who initiated a RPV/TDF/FTC co-formulation between April 2013 and March 2014 were included. Primary outcomes were the HIV-RNA viral load (copies/mL) and CD4 cell count (cells/mm^3^) at 6, 12 and 24 months. Reasons for a switch to RPV/TDF/FTC were evaluated through a standardized questionnaire. We also assessed discontinuation and reasons for discontinuation of RPV/TDF/FTC until October 30, 2015.

**Results:**

Of 644 individuals who started the RPV/TDF/FTC co-formulation, only 7.5% were treatment-naïve. At 24 months, viral suppression (HIV-RNA <50 copies/mL) was achieved in 100% and 96.7% of cART-naïve and cART-experienced patients respectively. The switch to RPV was mainly done for simplification (44.6%) and to overcome central nervous system toxicity symptoms due to efavirenz (24%). Six months after switch, 74.8% of patients reported an improvement of psycho-neurological symptoms with continued improvement at 12 months for almost 80%. However, one quarter of patients reported a discontinuation of RPV/TDF/FTC on October 30, 2015 after a median time of 18.4 months. Reasons for discontinuation included physician decision (5.3%) and side-effects (3.9%) mainly related to the central nervous system and to renal toxicity.

**Conclusion:**

The RPV/TDF/FTC co-formulation was safe and effective throughout 24 months of follow-up but barely prescribed for HIV-naïve patients. Despite excellent virological suppression among both treatment-naïve and -experienced patients, we observed a high rate of treatment discontinuation.

## Background

The combination of rilpivirine (RPV), tenofovir disoproxil (TDF) and emtricitabine (FTC) has demonstrated non-inferior efficacy in randomized controlled trials compared to efavirenz (EFV)-based regimens among HIV-infected, treatment-naïve patients with a baseline viral load of less than 10^5^ copies/mL [1–4]. RPV/TDF/FTC has also demonstrated efficacy in interventional [5] and observational [6–11] studies among treatment-experienced virologically suppressed patients switching to RPV. Current strategies with combined antiretroviral treatment (cART) focus on efficacy and safety in an aging HIV population with multiple comorbidities requiring additional treatments [12]. In this setting, RPV offers the advantages of few drug-drug interactions [13] and a favourable toxicity profile with a low incidence of grade 2–4 side-effects [14, 15]. In particular, fewer discontinuations due to central nervous system (CNS) adverse events in patients receiving RPV were reported compared to patients on EFV-based regimens [16]. Of note, EFV-related CNS toxicity has prompted its removal as a first-line regimen from several international guidelines in favour of integrase inhibitors, protease inhibitors [17] or RPV [18]. An improvement of lipid parameters was also observed with RPV compared to EFV or protease inhibitors, with a decrease in total and low-density lipoprotein cholesterol and triglycerides [4, 5].

RPV, co-formulated with TDF and FTC in a single tablet, was initially approved in Switzerland on April 1, 2013, for the treatment of HIV-naïve patients with a viral load less than 10^5^ copies/ml. Our experience suggested that the RPV/TDF/FTC co-formulation was used among virologically suppressed cART-experienced patients in a switch strategy, either to reduce adverse events due to the current regimen or for treatment simplification. We sought to evaluate the reasons for the prescription, as well as for discontinuation, of the RPV/TDF/FTC co-formulation in participants in the Swiss HIV Cohort Study (SHCS) after it entered the Swiss market. In addition, we assessed its effectiveness and safety at 6 (M6), 12 (M12) and 24 months (M24) post-initiation under routine clinical conditions.

## Methods

### Study design and patient population

We conducted a prospective analysis among all HIV-1 infected adults ≥18 years old participating in the SHCS who received at least one dose of the RPV/TDF/FTC co-formulation between April 1, 2013, and March 31, 2014. The SHCS is a multicentre prospective study established in 1988 and continuously enrolling HIV-infected individuals [19]. In brief, patients receive HIV care at one of the 7 outpatient clinics of the SHCS (Basel, Bern, Geneva, Lausanne, Lugano, St. Gallen, Zurich), at participating regional hospitals, or from collaborating private physicians. Follow-up visits are scheduled on a 6-monthly basis and include physical assessment, adherence check, review of medical conditions and drug prescriptions, as well as laboratory examinations [20]. Approximately 75% of all HIV infected patients on cART in Switzerland are followed within the SHCS network [21]. The scientific board of the SHCS approved this prospective analysis and all patients signed an informed consent form before enrolment in the SHCS.

### Variables

Primary outcomes assessed treatment effectiveness through the HIV-RNA viral load (copies/mL) and CD4 cell count (cells/mm^3^) measured at M6, M12 and M24 following initiation of the RPV/TDF/FTC co-formulation. We defined virological suppression as HIV-RNA <50 copies/mL. Secondary outcomes assessed treatment safety and used total cholesterol (mmol/L), high-density lipoprotein (mmol/L), triglycerides (mmol/L), alanine aminotransferase (UI/L), creatinine (μmol/L), and the estimated glomerular function rate (eGFR) (ml/min/1.73 m^2^) calculated according to the Modification of Diet in Renal Disease equation, as well as body mass index (kg/m^2^). Sociodemographic and clinical data were prospectively collected as part of the SHCS 6-monthly assessments at the time of initiation of the RPV/TDF/FTC co-formulation (referred to as baseline) and at M6, M12 and M24.

Reasons for switching to RPV/TDF/FTC were collected retrospectively. From January 1, 2015, onwards, the HIV Cohorts Data Exchange Protocol (HICDEP) coding [22] was used to document treatment switches in the SHCS database. However, before this date, treating physicians did not have the possibility to code regimen changes intended to simplify treatment. To obtain these data, we conducted a survey among treating physicians of all patients included in our study. We created a standardized questionnaire with a closed list of reasons for switching, which was first evaluated to assess its reliability among 6 physicians at the HIV unit of Geneva University Hospitals. The questionnaire was then sent to all physicians participating in the SHCS who had switched their patients to the RPV/TDF/FTC co-formulation. When physicians declared the reason for the switch as “toxicity, predominantly from CNS”, they were asked to give the exact reasons from a list of CNS-specific symptoms: i) symptoms of depression; ii) sleep disturbances/insomnia; iii) abnormal dreams; iv) dizziness/vertigo; v) fatigue/tiredness; and vi) other. Physicians were also required to document CNS symptoms at M6 and M12 after the switch as “worsening”, “stable”, “improvement”, “not available” or “other”.

In addition, we assessed a cross-section of patients who had discontinued the RPV/TDF/FTC co-formulation within M24 post-treatment initiation in both the treatment-naïve and -experienced groups and the reasons for discontinuation according to the HICDEP coding registered in the SHCS database.

### Statistical analysis

We differentiated two groups of patients: those who started RPV/TDF/FTC as a first-line regimen (cART-naïve patients) and those who switched from any cART regimen to RPV/TDF/FTC (cART-experienced patients).

We assessed if virological suppression were different across the 3 time-points (M6, M12 and M24) compared to baseline values among cART-experienced and among cART-naïve patients separately. To take into account the repetition of measurement in the same subject, we developed a generalized estimating equation using the binomial family, and exchangeable correlation structure. All models were adjusted for gender, age, history of AIDS event, HCV and HBV positivity. Then we used a linear multilevel model with a random effect on the patient to compare the evolution of the CD4 count across the 3 time-points compared to baseline values first among cART-experienced then among cART-naïve patients. We adjusted both models for the same variables described above plus the CD4 nadir (<50, 50–99, 100–199, 200–349, 350–499 and > = 500) and viral load (<= or >50 copies/mL). For safety parameters, we performed linear multilevel models with a random effect on the patient to compare the evolution of each parameter across the 3 time-points compared to baseline values. Again we performed the analyses separately in cART-experienced then in cART-naïve patients, and adjusted for gender, age, history of AIDS event, HCV and HBV positivity. We repeated the three models assessing the evolution of lipids (one model for cholesterol, triglycerides and finally HDL-cholesterol) across the 3 time-points compared to baseline values in the subgroup of cART-experienced patients previously treated with 2 NRTIs and one PI.

*P* values were calculated from generalized estimating equation (for viral load) and linear multilevel models (CD4, ALAT, creatinine, eGFR, cholesterol, triglycerides, HDL-cholesterol and BMI) comparing data at M6, M12 and M24 to baseline among cART-naïve then among cART-experienced patients, after adjustment for confounders described above.

Finally, we reported the proportions of cART-experienced and -naïve patients who discontinued the RPV/TDF/FTC co-formulation before October 30, 2015, and described the reasons.

All *P* values reported were two-sided and the level of significance was set at 0.05. Statistical analyses were conducted in STATA software, version 14 (StataCorp LP, College Station, TX, USA).

## Results

### Baseline characteristics of patients initiating RPV/TDF/FTC co-formulation

Between April 1, 2013, and March 31, 2014, 644 HIV-infected patients enrolled in the SHCS started the new RPV/TDF/FTC co-formulation. Most were male (70%; 451/644), Caucasian (73.9%; 476/644), and men who have sex with men (MSM) (47.7%; 307/644). Mean duration of HIV infection was 11 years (± standard deviation [SD]: 7.8) and mean age, 45.8 years (± 11.0 years); mean CD4 cell count at baseline and mean CD4 nadir were 637 (± 271) and 283 cells/mm^3^ (± 186), respectively. Among the 644 patients, 48 (7.5%) were cART-naïve at initiation of the RPV/TDF/FTC co-formulation, representing 10.5% of the total number of HIV-naïve patients enrolled in the SHCS and initiated cART during the same time period (*n* = 456). The baseline characteristics of patients initiating the co-formulation are shown in Table 1 and are presented for cART naïve and experienced patients. Among cART experienced patients, 44 (7.4%) were not fully virologically suppressed (HIV-RNA > 50 copies/mL, mean HIV-RNA 16629 copies/mL) at the time of the switch.

**Table 1:** Demographic, clinical, immunological and virological baseline characteristics of patients initiating RPV/TDF/FTC co-formulation between April 1, 2013, and March 31, 2014

Baseline characteristics	Total	cART-naïve patients	cART-experienced patients (*n* = 596)
	(*n* = 644)	(*n* = 48)	
Mean age, years (±SD, median)	45.8 (±11.1, 46)	42.3 (±11.3, 44.5)	46.1 (±11.0, 46)
Male gender, n (%)	451 (70.0)	41 (85.4)	410 (68.8)
HIV transmission group, n (%)			
MSM	307 (47.7)	29 (60.4)	278 (46.6)
Heterosexual	260 (40.4)	14 (29.2)	246 (41.3)
Intravenous drug use	44 (6.8)	2 (4.2)	42 (7.1)
Other^a^	14 (2.2)	2 (4.2)	12 (2.0)
Unknown	19 (2.9)	1 (2.0)	18 (3.0)
Ethnicity, n (%)			
White	476 (73.9)	37 (77.1)	439 (73.7)
Black	129 (20.0)	6 (12.5)	123 (20.6)
Hispano-American	18 (2.8)	4 (8.3)	14 (2.3)
Asian	20 (3.1)	1 (2.1)	19 (3.2)
Other	1 (0.2)	0 (0)	1 (0.2)
Mean duration of HIV infection in years (±SD, median)	11.0 (±7.8, 9.8)	2.6 (±3.3, 1.5)	11.7 (±7.7, 10.6)
History of AIDS disease, n (%)	91 (14.1)	0 (0)	91 (15.3)
Mean baseline^b^ CD4 count, cells/mm^3^ (±SD, median)^c^	637 (±271, 606)	478 (±176, 473)	650 (±273, 620)
Mean nadir CD4 count, cells/mm^3^ (±SD, median)^d^	283 (±186, 261.5)	447 (±157, 430)	270 (±182, 247)
Baseline^b^ HIV-RNA < 50 copies/mL, n (%)^c^	552 (86.1)	2 (4.2)^e^	549 (92.6)
HBV co-infection (positive AgHBs), n (%)	32 (5.0)	0 (0)	32 (5.4)
HCV co-infection (positive HCV-RNA), n (%)	35 (5.4)	1 (2.1)	34 (5.7)

^a^Other = blood products, perinatal transmission, other

^b^Baseline = at time of initiation of or switch to RPV/TDF/FTC co-formulation

^c^Missing data (*n* = 641, 48/48 available in naïve, 593/596 in experienced)

^d^Missing data (*n* = 640, 47/48 available in naïve, 593/596 in experienced)

^e^Two cART-naïve patients started treatment with an HIV-RNA <50 copies/mL: one because of anxiety related to HIV-infection, the other was taking a combination of tenofovir and emtricitabine intermittently without his physician knowledge. We still considered the latest as treatment-naïve of cART regimen

cART combined antiretroviral treatment, MSM men who have sex with men, SD standard deviation, HBV hepatitis B virus, HCV hepatitis C virus

### Reasons for a switch to RPV/TDF/FTC in cART-experienced patients

The study questionnaire was sent to the SHCS treating physicians in November 2014. At that time, we had identified 598 patients who were switched to RPV/TDF/FTC during the study period. Four patients were excluded as one did not receive the RPV/TDF/FTC co-formulation and 3 were cART-naïve according to the treating physicians. The non-response rate was 9.5% (57/598 questionnaires). For these 57 patients, as well as two additional patients identified later and without a questionnaire, we retained the reason for the switch as registered in the SHCS database. The mean duration of cART treatment at the time of switch was 8.7 years (SD: ±5.9). Before switching, 47.8% (285/596) of cART-experienced patients were on a non-nucleoside reverse transcriptase inhibitor (NNRTI)-based regimen, 26.2% (156/596) were on a protease inhibitor-based regimen, and 9.7% (58/596) were on an integrase inhibitor-based regimen. Of those on a NNRTI regimen, two thirds (192/285) were on EFV. Regimens before switch and reasons for switch are presented in Table 2. Treatment simplification (266/596; 44.6%) and CNS toxicity (143/596; 24.0%) were the two main reasons for switch.

**Table 2:** Previous regimens at time of switch and main reasons for switch among the 596 cART-experienced patients initiating a RPV/TDF/FTC co-formulation

	*N* (%)
ART regimen at switch	
2 NRTIs + EFV	192 (32.2)
2 NRTIs + NVP or ETV	93 (15.6)
2 NRTIs +1 PI	156 (26.2)
2 NRTIs +1 INSTI	58 (9.7)
Triple nuke regimen	44 (7.4)
Other	29 (4.9)
Unknown	24 (4.0)
Main reasons for switch	
Simplification	266 (44.6)
CNS toxicity	143 (24.0)
Physician decision	46 (7.7)
Gatrointestinal/liver toxicity	42 (7.0)
Abnormal fat distribution/dyslipidemia/concern of cardiovascular disease	38 (6.4)
Other toxicities (including endocrine, haematological, kidney, muscle, skin)	26(4.4)
Patient wish/decision	20 (3.4)
Drug interaction	6 (1.0)
Treatment failure	1 (0.2)
Unknown	8 (1.3)

RPV/TDF/FTC rilpivirine/tenofovir/emtricitabine, cART combined antiretroviral treatment, NRTI nucleoside reverse transcriptase inhibitor, NNRTI non-nucleoside reverse transcriptase inhibitor, EFV efavirenz, NVP nevirapine, ETV etravirine, PI protease inhibitor, INSTI integrase strand transfer inhibitor

CNS toxicity was the reason for the switch for 126 patients on an EFV-based regimen (65.6%; 126/192) with a description of symptoms available for 123 patients (Table 3). Among these, 60.2% (74/123 patients) had more than one symptom, and a total of 197 CNS adverse events were reported: insomnia/sleep disturbances (26.9%; 53/197); abnormal dreams (18.8%; 37/197); depression (17.3%; 34/197); dizziness (15.2%; 30/197); fatigue/tiredness (13.7%; 27/197); and other reasons (8.1%; 16/197). Six months after the switch from EFV to RPV, 74.8% (92/123) of patients reported an improvement of CNS symptoms, 14.6% (18/123) reported a stable condition and 3.2% (4/123) described worsening CNS side effects. Continuous improvement in CNS symptoms at M12 was reported for 78.3% of patients for whom we had data (72/92) while condition remained stable for 6.5% (6/92) of patients.

**Table 3:** Central nervous system (CNS) adverse events experienced by the 123 patients on EFV-based regimens reporting CNS symptoms prior to switch and change over time on RPV/TDF/FTC co-formulation

	*N* (%)
CNS adverse events on previous EFV-based regimens	197 (74 patients with > 1 symptoms)
Insomnia/sleep disturbances	53 (26.9%)
Abnormal dreams	37 (18.8%)
Symptoms of depression	34 (17.3%)
Dizziness/vertigo	30 (15.2%)
Fatigue/tiredness	27 (13.7%)
Other	16 (8.1%)
Change in CNS symptoms reported by patients at 6 months after switch from EFV to RPV	123
Worsening condition	4 (3.3%)
Stable condition	18 (14.6%)
Improved condition	92 (74.8%)
Other/unknown	9 (7.3%)
Further change in CNS symptoms at 12 months after switch from EFV to RPV in those who improved at M6	92
Worsening condition	6 (6.5%)
Stable condition	72 (78.3%)
Improved condition	13 (14.1%)
Other/unknown	1 (1.1%)

*CNS* central nervous system, *EFV* efavirenz, *RPV* rilpivirine, *M6* month 6

### Effectiveness and safety of the RPV/TDF/FTC co-formulation

**Fig. 1:**
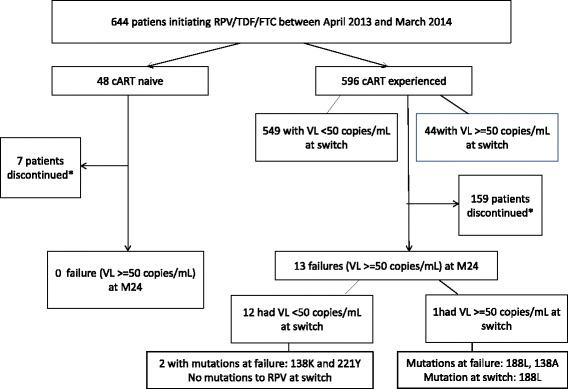
Flowchart of patients initiating RPV/TDF/FTC co-formulation, discontinuation and treatment failures. *Discontinuation for other reasons than virological failure Vl viral load, cART combined antiretroviral therapy

Effectiveness and safety variables over time are presented both for treatment-naïve and –experienced patients in Table 4. Viral suppression (HIV-RNA < 50 copies/mL) was achieved among 93.8%, 97.6% and 100% of the cART naive patients at M6, M12 and M24 respectively (*P* < 0.001). At M24, 13 patients in the cART experienced group did not meet the criteria for virological suppression, i.e. HIV-RNA <50 /mL. These 13 patients were switched mostly from a PI regimen (46%, 6/13) and from a NNRTI regimen (38.5%, 5/13). Genotype was available for the 6 patients failing with an HIV-RNA ≥ 200 copies/mL: 3 patients had a mutation conferring resistance to RPV at the time of virological failure, either 138A/K, 188 L or 221Y mutation. One of these 3 patients was not fully suppressed and already had developed a 188 L mutation at the time of the switch to RPV. Figure 1 shows the number of cART naive and experienced patients discontinuating RPV/TDF/FTC combination for virological failure or other reasons.

**Table 4:** Efficacy and safety parameters (mean values ±standard deviation, median) at M6, M12 and M24 after initiation (baseline) of a RPV/TDF/FTC co-formulation among cART-naïve and cART-experienced patients

	*Baseline* ^*†*^	*M6* ^*††*^	*M12* ^*†††*^	*M24* ^*††††*^	P value over time*
Viral load (copies/mL), n (%)					
cART-naïve					
<50	2 (4.2)^¥^	45 (93.8)	41 (97.6)	26 (100)	<0.001
> = 50	46 (95.8)	3 (6.2)	1 (2.4)	0 (0)	
cART-experienced					
<50	549 (92.6)	562 (96.2)	514 (97.5)	380 (96.7)	0.002
> = 50	44 (7.4)	22 (3.8)	13 (2.5)	13 (3.3)	
CD4 count (cells/mm^3^), ±SD, median					
cART-naïve	478 (±176, 473)	622 (±204, 602)	621 (±201, 581)	704 (±248, 649)	0.004
cART-experienced	650 (±273, 620)	676 (±285, 643)	681 (±286, 633)	697 (±363, 660)	0.001
ALAT (UI/L), ±SD, median					
cART-naïve	31 (±18, 28)	31 (±16, 30)	31 (±16, 28)	30 (±14, 32)	0.578
cART-experienced	39 (±49, 29)	38 (±42, 29)	35 (±25, 29)	36 (±63, 28)	0.284
Creatinine (μmmol/L), ±SD, median					
cART-naïve	94 (±82, 82)	88 (±15, 86)	88 (±15, 86)	91 (±13, 89)	0.887
cART-experienced	78 (±16, 77)	85 (±18, 85)	86 (±18, 86)	85 (±21, 83)	<0.001
eGFR (mL/min/1.73m^2^), ±SD, median					
cART-naïve	98 (±25, 100)	90 (±20, 89)	91 (±21, 91)	90 (±26, 86)	0.008
cART-experienced	100 (±25, 96)	90 (±28, 86)	88 (±23, 85)	89 (±26, 87)	<0.001
Cholesterol (mmol/L), ±SD, median					
cART-naïve	4.7 (±1.0, 4.8)	4.5 (±1.0, 4.4)	4.5 (±1.0, 4.4)	NA	0.270
cART-experienced	5.1 (±1.0, 5.0)	4.7 (±1.0, 4.6)	4.7 (1.0, 4.6)	NA	<0.001
Triglycerides (mmol/L), ±SD, median					
cART-naïve	1.5 (±1.1, 1.2)	1.5 (±1.1, 1.3)	1.5 (±0.9, 1.4)	1.3 (±0.6, 1.2)	0.347
cART-experienced	1.6 (±1.3, 1.3)	1.4 (±0.9, 1.2)	1.4 (±0.9, 1.2)	1.4 (±0.9, 1.2)	<0.001
HDL-cholesterol (mmol/L), ±SD, median					
cART-naïve	1.2 (±0.4, 1.2)	1.2 (±0.3, 1.2)	1.1 (±0.3, 1.2)	NA	0.195
cART-experienced	1.3 (±0.4, 1.3)	1.3 (±0.3, 1.2)	1.2 (±0.3, 1.2)	NA	<0.001
BMI (kg/m^2^), ±SD, median					
cART-naïve	23.6 (±5.6, 22.6)	24.2 (±3.8, 23.7)	24.2 (±3.9, 23.8)	22.9 (±1.0, 22.9)	0.006
cART-experienced	24.9 (±5.3, 24.4)	24.7 (±4.0, 24.3)	24.7 (±3.9, 24.3)	24.8 (±4.6, 24.0)	0.096

^†^Missing data (ASAT 37/48 available in naïve patients, 565/596 in experienced; ALAT 37/48 available in naïve patients, 576/596 in experienced; creatinine and eGFR 33/48 available in naïve patients, 463/596 in experienced; triglycerides 36/48 available in naïve patients, 540/596 in experienced; cholesterol 36/48 available in naïve patients, 542/596 in experienced; HDL 36/48 available in naïve patients, 530/596 in experienced; weight 48/48 available in naïve patients, 574/596 in experienced; waist and hip 48/48 available in naïve patients, 563/596 in experienced; BMI 48/48 available in naïve patients, 574/596 in experienced)

^††^Missing data (ASAT/ALAT/Cholesterol 48/48 available in naïve patients, 583/596 in experienced; creatinine/eGFR/triglycerides 48/48 available in naïve patients, 578/596 in experienced; HDL/weight/BMI 48/48 available in naïve patients, 582/596 in experienced; waist and hip 48/48 available in naïve patients, 580/596 in experienced)

^†††^Missing data (ASAT/ALAT 42/48 available in naïve patients, 527/596 in experienced; creatinine 42/48 available in naïve patients, 521/596 in experienced; eGFR 41/48 available in naïve patients, 517/596 in experienced; triglycerides 41/48 available in naïve patients, 522/596 in experienced; cholesterol 41/48 available in naïve patients, 526/596 in experienced; HDL 41/48 available in naïve patients, 525/596 in experienced; weight 44/48 available in naïve patients, 513/596 in experienced; waist 44/48 available in naïve patients, 511/596 in experienced; hip 44/48 available in naïve patients, 510/596 in experienced; BMI 44/48 available in naïve patients, 513/596 in experienced)

^††††^Missing data (ALAT 15/48 available in naïve patients, 239/596 in experienced; creatinine 14/48 available in naïve patients, 236/596 in experienced; eGFR 14/48 available in naïve patients, 235/596 in experienced; triglycerides 14/48 available in naïve patients, 232/596 in experienced; cholesterol 0/48 available in naïve patients, 0/596 in experienced; HDL 0/48 available in naïve patients, 0/596 in experienced; weight 4/48 available in naïve patients, 158/596 in experienced; waist 4/48 available in naïve patients, 151/596 in experienced; hip 4/48 available in naïve patients, 151/596 in experienced; BMI 4/48 available in naïve patients, 158/596 in experienced)

*P-values from generalized estimating equation (for viral load) and linear multilevel models (CD4, ALAT, creatinine, eGFR, cholesterol, triglycerides, HDL-cholesterol and BMI) comparing data at M6, M12 and M24 to baseline among cART-naïve and cART-experienced patients separately, after adjustment for main confounders

RPV/TDF/FTC rilpivirine/tenofovir/emtricitabine, cART combined antiretroviral treatment, ASAT aspartate aminotransferase, ALAT alanine aminotransferase, eGFR glomerular filtration rate, HDL high-density lipoprotein, BMI body mass index

^¥^Two cART-naïve patients started treatment with an HIV-RNA <50 copies/mL: one because of anxiety related to HIV-infection, the other was taking a combination of tenofovir and emtricitabine intermittently without his physician knowledge

CD4 count significantly increased at M6, at M12 and at M24 compared to baseline values in both cART naive and experienced patients (Table 4).

For safety, we found that creatinine values significantly increased across the 3 time-points, M6, M12 and M24, in cART-experienced patients (*P* < 0.001) but not in cART-naive patients. However, the creatinine clearance (or eGFR) decreased over time in both groups. Total cholesterol, triglycerides and HDL-cholesterol significantly decreased over time among cART-experienced patients (Table 4). When considering the subgroup of patients on PI-based regimen before the switch to RPV/TDF/FTC, cholesterol decreased significantly at M6 (mean 4.64 ± 0.96 SD, median 4.5) and M12 (4.64 ± 0.94, 4.55) compared to baseline values (5.09 ± 0.98 SD, median 5.01, *p* < 0.001). Similarly, HDL-cholesterol decreased significantly at M6 (1.25 ± 0.37 SD, median 1.17) and M12 (1.26 ± 0.38 SD, median 1.20) compared to baseline values (1.31 ± 0.40 SD, median 1.20, *p* = 0.004). Triglycerides decreased significantly across time (M6: 1.41 ± 1.01 SD, median 1.20; M12: 1.40 ± 1.07 SD, median 1.12) compared to baseline (1.73 ± 1.21 SD, median 1.50, *p* < 0.001) but at M24 values were not significantly different from those at baseline (1.50 ± 1.20, 1.10, *p* = 0.516).

### Follow-up on October 30, 2015

On October 30, 2015, we assessed all patients who initiated a RPV/TDF/FTC co-formulation between March 2013 and April 2014. The median follow-up time since RPV/TDF/FTC initiation was 18.4 months (interquartile range: 14.0–21.9). Overall, 166/644 (25.8%) patients had discontinued the RPV/TDF/FTC co-formulation on October 30, 2015 (Table 5).

**Table 5:** cART-experienced and cART-naïve RPV/TDF/FTC patients who discontinued treatment between April 2014 and October 2015; reasons for discontinuation and next cART regimen

	Number (%) of patients	
	cART-naïve RPV/TDF/FTC (*n* = 48)	cART-experienced RPV/TDF/FTC (*n* = 596)
Number of patients discontinuating	7 (14.5%)	159 (26.7%)
Reasons for discontinuation		
Treatment failure	1 (2.1%)	5 (0.8%)
Physician decision	2 (4.2%)	34 (5.7%)
Patient request	1 (2.1%)	11 (1.8%)
CNS toxicity	0 (0%)	10 (1.7%)
Availability of more effective treatment	1 (2.1%)	7 (1.2%)
Kidney toxicity	0 (0%)	7 (1.2%)
Other toxicities (including gastrointestinal, liver, endocrine, dyslipidemia, abnormal fat distribution)	0 (0%)	8 (1.3%)
Other causes (pregnancy, enrolment in drug trial, drug interaction, prevention of side-effects, non-compliance)	0 (0%)	8 (1.3%)
Unspecified causes	0 (0%)	9 (1.5%)
Unknown	2 (4.2%)	60 (10.1%)
Next treatment regimen after discontinuation		
INSTI-based		
DTG/ABC/3TC co-formulation	2 (4.2%)	25 (4.2%)
EGV/COB/TDF/FTC co-formulation	2 (4.2%)	16 (2.7%)
Other DTG-based regimen	1 (2.1%)	29 (4.9%)
Other EVG-based regimen	0 (0%)	3 (0.5%)
RAL-based regimen	1 (1.2%)	13 (2.2%)
PI-based	0 (0%)	18 (3.0%)
NNRTI-based	0 (0%)	24 (4.0%)
Other	0 (0%)	4 (0.7%)
Unknown	1 (1.2%)	27 (4.5%)

cART combined antiretroviral treatment, RPV/TDF/FTC rilpivirine/tenofovir/emtricitabine, CNS central nervous system, INSTI integrase strand transfer inhibitor, DTG dolutegravir, ABC abacavir, 3TC emtricitabine, EGV elvitegravir, COB cobicistat, PI protease inhibitor

Besides treatment failure as described above, the most common reasons provided for discontinuation were unknown (62/644; 9.6% of all patients); physician decision with no reason specified (34/644; 5.3%); patient request (12/644; 1.9%); and CNS toxicity (10/644; 1.6%). Among the 10 patients who discontinued RPV/TDF/FTC co-formulation for CNS side effects, all were cART-experienced patients with 3 of them previously receiving EFV and who had already switched to RPV/TDF/FTC for the same reason. There were no deaths among the study population. Of the 166 discontinuations, more than half (*n* = 92; 55.4%) had discontinued the RPV/TDF/FTC co-formulation for an integrase inhibitor-based regimen. Of these switches, 44.9% (45/92) switched to co-formulations including dolutegravir or elvitegravir.

## Discussion

We demonstrated that rilpivirine, tenofovir disoproxil and emtricitabine in a fixed dose combination is an effective cART regimen in both treatment-experienced and -naïve patients under routine clinical conditions, although RPV was rarely used in naïve patients. Ninety-six percent of treatment-experienced patients and 100 % of treatment-naïve patients were virologically suppressed at M24. Our findings are similar to clinical trials that showed 84–86% of virological success for RPV/TDF/FTC at M12 [3, 4] and 84% at M24 [23] in naïve patients, and 85.8% of virological success for RPV at M12 in experienced patients [5]. Our results are also consistent with observational studies assessing a treatment switch to RPV (>93% of virological success of RPV at M12) [6, 7], but none of them assessed the use of RPV among naïve patients under routine clinical care. Only one observational study reported a lower proportion of virological suppression at M12; results were explained by an inappropriate switch to a RPV-containing regimen in patients with previous virological failure and by missing data [11]. In our study, 13 treatment-experienced patients had an HIV-RNA above 50 copies/ml at M24. We were able to obtain genotypic data in the 6 patients failing with an HIV-RNA ≥ 200 copies/mL: 3 patients carried 138A/K, 188 L or 221Y mutations at the time of failure, mutations that are known to confer resistance to RPV [24]. One patient had a detectable viral load and had already developed a 188 L mutation at the time of switch and should have not received the RPV/TDF/FTC combination. The rate of confirmed treatment failure was therefore very low in our study and detectable HIV-RNA may translate blips in viral load or poor adherence rather than virological failure.

Among cART-experienced patients, the main reasons for a switch to the newly marketed RPV/TDF/FTC co-formulation were treatment simplification and CNS toxicity. The proportion of patients switching to RPV for simplification is slightly lower than in other observational studies [6, 11]. Unlike previous reports [6, 7, 11], we accurately assessed the reasons for a switch to RPV with a dedicated detailed questionnaire. Due to the retrospective manner and a non-response rate of 9.5% to our standardized questionnaire, it is possible that the simplification reason was underreported or rather stated as “physician decision” or “patient request”. Among those who switched because of CNS toxicity, most were on an EFV-based regimen. Symptoms did improve in approximately 75% of patients 6 months after the switch with a continuous improvement at M12. Moreover, only 3 patients switched from EFV to RPV/TDF/FTC later discontinued RPV co-formulation for persisting CNS side effects. While there was no change in neurocognitive functions after EFV replacement for a PI drug in a small controlled trial enrolling 16 patients [25], another observational study confirmed improvement in neurological side-effects in almost 50% of patients switching from an EFV-based regimen to RPV [6]. Switch from EFV to RPV seems therefore a reasonable and sustainable option for patients experiencing CNS side effects on EFV.

Overall, the RPV/TDF/FTC co-formulation was safe and well tolerated in most patients. As shown in previous studies [6, 7, 11], we observed a significant increase in creatinine over time among our study population, but this difference was not clinically relevant. Rilpivirine is known to inhibit the creatinine transporter in the proximal renal tubule [13]. Similarly to other studies [6, 7, 11], we did observe a significant change in the lipid profile of treatment-experienced patients after the switch to RPV/TDF/FTC. This was also true for the subset of patients previously on PI. However, there was no effect of RPV/TDF/FTC on lipid profile over time among cART-naïve patients; this may be due to the small number of treatment naïve subjects included and the lack of power to detect an effect.

Despite good results for the effectiveness and tolerability of the RPV/TDF/FTC co-formulation, a quarter of patients changed their ART regimen, mainly upon physician decision, after a mean time of 18.4 months. This is much shorter than the reported mean duration of patients on first- and second-line newer cART regimens (4.6 and 3.9 years, respectively) in the 2008–2011 period as described in the HIV Outpatient Study [26]. We hypothesized that there is a low confidence in the RPV genetic barrier among treating physicians [27], particularly when compared to newer once-daily, inhibitor-based co-formulation regimens, the latest being marketed in Switzerland only 2 years after the RPV/TDF/FTC co-formulation. The need for a fatty meal intake may be also a barrier to the large-scale prescription of this regimen although the acceptability of food constraint was not formally assessed in our study. Finally, less than 5% of patients discontinued the RPV/TDF/FTC co-formulation due to side-effects, mainly CNS-related symptoms and kidney toxicity. Neuropsychiatric adverse events due to RPV were described in up to 27% of naïve patients receiving 48 weeks of RPV in clinical trials, but discontinuation was rare [16]. In our study, discontinuation of RPV for CNS toxicity (1.6%) was much lower than discontinuation of EFV for the same reason (65.6%). It is also lower than discontinuation rates reported on dolutegravir in routine clinical settings, which ranged between 3.4% and 6% [28].

Although we included a small number of cART-naïve patients, our study is the first to report on treatment outcomes up to 24 months under routine clinical conditions in this population. This may provide the necessary confidence to clinicians to prescribe the RPV/TDF/FTC co-formulation to HIV-naïve patients if the virological criteria are met, and providing that the patient consents to comply with the dietary restrictions.

Our study has several limitations. First, the standardized questionnaire on the reasons for a switch to the RPV/TDF/FTC co-formulation was retrospective and therefore subject to recall bias, particularly regarding the possible over-reporting of CNS toxicity symptoms and their improvement after a switch from EFV to RPV. Second, the study population was mainly Caucasian, male, and MSM, which renders more difficult the generalizability of our results to under-represented transmission groups. Finally, we did not compare initiation to RPV/TDC/FTC co-formulation to starts and switches to alternative regimens during the same time period.

## Conclusions

The use of RPV is safe and effective under routine clinical conditions, both in a switch strategy and in naïve patients. Our study supports policy changes made in Switzerland in October 2014 to use RPV for cART-experienced virologically suppressed patients. RPV demonstrates a favourable neurological toxicity profile with most patients experiencing CNS side effects on EFV improving after a switch to RPV and very low discontinuation rate for CNS events when compared to EFV or integrase inhibitor. Future research should focus on comparing the RPV/TDF/FTC co-formulation with newer available regimens containing integrase inhibitors in a switch strategy, including an assessment of cost-effectiveness. The reasons for early discontinuation of RPV/TDF/FTC co-formulation based on physician decision in the SHCS should also be further explored.

## Abbreviations

AIDS: Acquired immunodeficiency syndrome
cART: Combined antiretroviral therapy
CNS: Central nervous system
EFV: Efavirenz
eGRF: Estimated glomerular function rate
FTC: Emtricitabine
HBV: Hepatitis B virus
HCV: Hepatitis C virus
HICDEP: HIV Cohorts Data Exchange Protocol
HIV: Human immunodeficiency virus
M12: Month 12
M24: Month 24
M6: Month 6
MSM: Men who have sex with men
RPV: Rilpivirine
SHCS: Swiss HIV cohort study
TDF: Tenofovir
